# A Systematic Review of Open and Minimally Invasive Surgery for Treating Recurrent Hallux Valgus

**DOI:** 10.1055/s-0042-1759812

**Published:** 2022-12-21

**Authors:** Arun Nair, Matthew Bence, Jawaad Saleem, Azka Yousaf, Lena Al-Hilfi, Kumar Kunasingam

**Affiliations:** 1 Department of Trauma & Orthopaedics, Croydon University Hospital, Croydon, United Kingdom

**Keywords:** MIS, minimally invasive hallux valgus, bunion surgery, revision hallux valgus

## Abstract

**Background** Despite advancements in primary correction of hallux valgus (HV), significant rates of reoperation remain across common techniques, with complications following primary correction up to 50% according to some studies. 1 This study explored different methods of surgery currently used in treating HV recurrence specifically (for which literature on the subject has been limited), evaluating open and adapted minimally invasive surgical (MIS) primary techniques used for revision.

**Methods** In December 2020, literature search for both open and MIS surgical techniques in HV revision was conducted using PubMed, EMBASE, and MEDLINE library databases.

**Results and Conclusion** Of initial 143 publications, 10 were finally included for data synthesis including 273 patients and 301 feet. Out of 301 feet, 80 (26.6%) underwent revision with MIS techniques (involving distal metatarsal osteotomies). Those undergoing grouped MIS revisions had an average improvement of 38.3 in their American Orthopaedic Foot and Ankle Society score, compared to 26.8 in those using open techniques. Revision approaches using grouped MIS techniques showed a postoperative reduction in intermetatarsal angle and HV angle of 5.6 and 18.4 degrees, respectively, compared to 15.5 and 4.4 degrees, respectively, for open techniques. There are, however, limitations in the current literature on MIS techniques in revision HV surgery specifically. MIS techniques grouped did not show worse outcomes or safety concerns compared to open techniques.

Hallux valgus (HV) is a common chronic foot condition caused by lateral deviation of the great toe and prominence of the first metatarsal head; affecting as many as a quarter of adults aged above 40 years old in the U.K. 1 The deformity can be progressive, with resultant worsening inflammation of the metatarsophalangeal (MTP) joint causing significant pain and impairment of health-related quality of life. 2 3 Subsequent operative correction of HV causes significant improvements in patient-reported outcomes measures (PROMs) such as EuroQol 5 Dimension, 36-Item Short Form Health Survey, and foot and ankle specific scores. 4

Methods of operative correction involve osteotomy, resection arthroplasty, soft tissue procedure, arthrodesis, and combinations of these techniques, often with concomitant bunionectomy. Over 150 variants of convention open operative techniques have been described for primary correction of HV, reflecting the fact that HV is not a single deformity, but a composite of bony and soft tissue deformities centered around the first ray. 5 Within the last decade the traditional operative management has been adapted, making use of minimally invasive surgical (MIS) techniques. 6 7 8

Despite advancements in the primary correction of HV, significant rates of reoperation remain across common techniques. 9 Complications including recurrence, infection, and hallux varus, may affect as many as 50% of patients undergoing primary correction of HV. 10 Recurrence of HV is one of the most common adverse events, affecting between 4 and 25% of cases. 11 Recurrence of HV is also associated with worsening of patient outcome scores, most notably through chronic pain. 10 12 Newer percutaneous techniques for treating HV have demonstrated recurrence rates as high as 15.2% following primary correction. 13

The purpose of this study is to perform a systematic review exploring current management techniques for recurrent HV and corresponding outcomes. Particular attention was paid toward papers describing newer MIS techniques to ascertain whether the results would be comparable with established traditional operative treatments for recurrent HV. Papers were included examining both patient-reported outcomes as well as objective measurements of the radiographic parameters used to assess HV.

## Materials and Methods

### Search Strategy

**Fig. 1:**
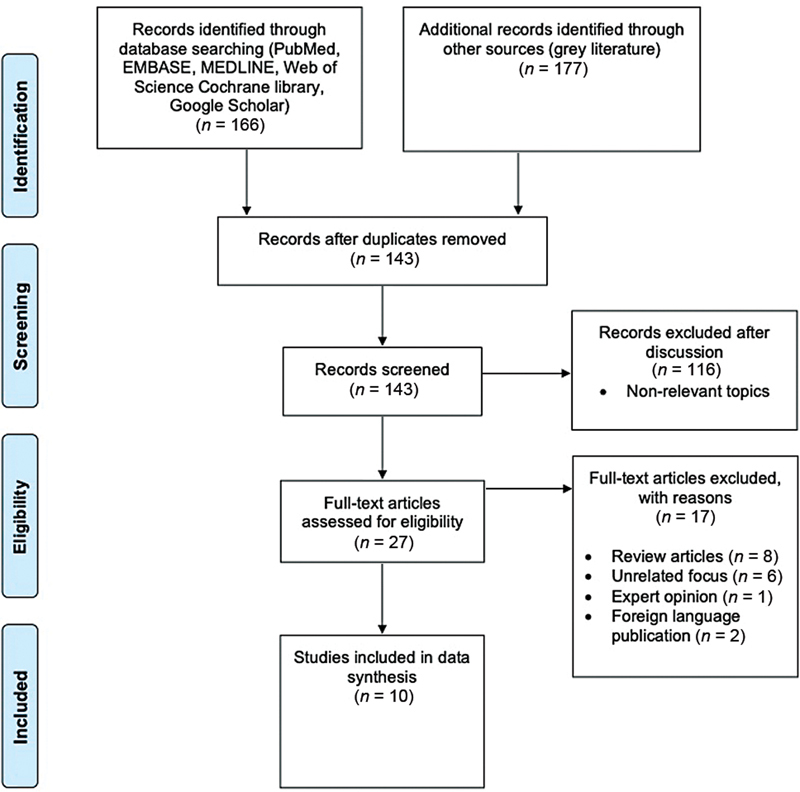
Preferred Reporting Items for Systematic Reviews and Meta-Analyses (PRISMA) flowchart detailing included and excluded studies.

Search strategy was performed according to the Preferred Reporting Items for Systematic Reviews and Meta-Analyses guidelines ( Fig. 1 ).

On December 8, 2020, a literature search for both open and MIS operative techniques in HV revision was conducted using PubMed, EMBASE, MEDLINE, Web of Science, Google Scholar, and Cochrane library databases using the following search strategy: (hallux valgus OR bunion) AND (revision OR revise OR recurrence OR recurrent) AND (minimally invasive surgery OR minimally invasive OR MIS) OR (revision hallux valgus). Combinations of keywords including “percutaneous” and “arthroscopic” were also used. Publication date range was from the year 2000 to date of literature search as above.

Gray literature was searched manually (British Library EThOS, OATD, DART Europe, OpenGrey, OCLC, NIH Clinical Trials, TRIP medical database, Bone and Joint Publishing: Orthopaedic Proceedings)—no relevant articles were identified from these sources during the screening process.

Following removal of duplicates, 143 publications were produced from the initial search of databases. After screening and discussion, 27 full text articles were assessed for eligibility. References were also screened and articles from nonpeer reviewed journals were removed. Ten articles met the criteria for inclusion. 14 15 16 17 18 19 20 21 22 23

### Inclusion and Exclusion Criteria

All studies included were English language publications of patients who had undergone revision surgery following primary HV correction surgery. Included studies required reporting of data points for both primary and revision surgery—this included patient demographics, operative features, outcomes, and complications. Review articles, expert opinion cases, and single case reports were also excluded.

## Results

### Demographic Data

Using the Oxford Centre for Evidenced-Based Medicine criteria, 8 studies were classified as level 4 (case series) and 2 studies as level 3 (case–control studies). 24 All 10 studies were single-center based including a total of 273 patients and 301 feet ( Table 1 ). Age range for included patients was between 21 and 89, with the majority of patients in their late 40s. Women formed the vast majority of patients at 243 (89%) compared to 30 men (11%). The median time between primary HV corrective procedure and revision ranged from 10 months to 38 years. The median time for patient follow-up ranged from 1 and 89 months. Two studies detailed patients' comorbidities including smoking history (5 patients), diabetes (3 patients), inflammatory arthritis (2 patients), peripheral neuropathy (1 patients), and pseudogout (1 patients), whereas presence of comorbidity was not specified in the other 8 articles.

**Table 1:** Demographic characteristics in patients for included studies

No	Article (year)	Design	Level of evidence	No. of patients (feet)	Median age – years (range)	Male/Female	Median time between index and revision surgery - years (range)	Median follow up period - months (range)	Associated comorbidities
1	Magnan et al (2019)	Retrospective case series	4	26 (32)	54.2 (33–68)	1/25	9.7 (2–23)	9.8 (2.4–15.2)	NA
2	Scala et al (2020)	Retrospective case–control study	3	52 (54)	49 (22–76)	6/46	NA	2.5 (1–5.5)	NA
3	Ellington et al (2011)	Retrospective case series	4	23 (25)	NA	1/24	7.6 (0.8–19.3)	31.6 (12–60)	NA
4	Bock et al (2010)	Retrospective case series	4	35 (39)	58.8 (33–78)	1/34	9.7 (1–23)	42 (24–89)	NA
5	Grimes and Coughlin (2006)	Retrospective case series	4	29 (33)	62 (29–89)	4/25	10.7 (0.8–35)	8 (1–22)	Diabetes (3), inflammatory arthritis (2), peripheral neuropathy (1), pseudogout (1)
6	Kannegieter and Kilmartin (2011)	Retrospective case series	4	5 (NA)	55 (NA)	0/5	4 (1.2–8.8)	38 (3–69)	NA
7	Rose et al (2014)	Retrospective case series	4	31 (36)	53.4 (26–76)	3/28	10 (1–31)	3.9 (1–5)	NA
8	Vienne et al (2006)	Retrospective case series	4	20 (22)	63.1 (48–84)	3/17	NA	34 (24–48)	NA
9	Coetzee et al (2003)	Retrospective case series	4	24 (26)	37 (21–57)	10/14	NA	21.6 (6–36)	Smoking history (5)
10	Machacek et al (2004)	Retrospective case–control study	3	28 (29)	64 (49–78)	2/26	13 (2–38)	36 (24–76)	NA

### Types of Primary Surgery

The index procedures for HV correction are summarized in Table 2 . Out of the 301 feet, 195 (64.8%) underwent first metatarsal/phalangeal osteotomies, 60 (19.9%) underwent resection arthroplasty, and 19 (6.3%) underwent soft tissue procedures. Of the remaining 27 feet (9%), all but one foot (which had no primary procedure documented) underwent simple bunionectomies alone for primary correction of HV. All 10 studies stated exclusion criteria for revision surgery which varied greatly—including diabetes, peripheral vascular disease, inflammatory arthritis, severe degeneration or stiffness of joint, and minimum follow-up time. Such variation emphasized the limited role for meta-analysis in this study. Of the 195 osteotomy procedures performed, 23 (11.8%) were identified involving MIS techniques (distal first metatarsal osteotomies)—with percutaneous HV correction using an Reverdin–Isham osteotomy (9) and Bosch–Magnan osteotomy (14). 15

**Table 2:** Categories of index procedures

Category	Procedure
First metatarsal/phalangeal osteotomy	Austin, Akin, Chevron, proximal osteotomy, Lamprecht-Kramer, Scarf, PDO, Reverdin–Isham, Bosch–Magnan
Resection/Interposition arthroplasty	Keller
Soft tissue procedures	McBride tissue release, medial capsule retention
Arthrodesis	Lapidus procedure, MTP arthrodesis
Bunionectomy (Exostectomy)	–

Abbreviations: MTP, metatarsophalangeal; PDO, percutaneous distal osteotomy.

### Types of Revision Surgery

Out of the 301 feet, 86 (28.6%) underwent revision with MIS techniques—with 32 undergoing a distal first metatarsal osteotomy 14 and 54 undergoing a modified subcapital metatarsal osteotomy (MSMO). 15 Open revision techniques included 80 scarf osteotomies (26.6%), 51 Lapidus procedures (16.9%), and 84 first MTP joint arthrodesis (27.9%). Four studies indicated if their patients/feet included had had multiple revisions following primary correction of HV prior to the planned revision procedure as part of their study—this included 2 bunionectomies prior to MSMO and 4 bunionectomies prior to first MTP arthrodesis.

### Reasons for Revision

Four of the studies included varying radiological evidence in determining reason for revision surgery—using measurements of HV angle (HVA), intermetatarsal angle (IMA), and distal metatarsal articular angle (DMAA), 7 studies included qualitative indications for revision including failure of conservative treatment, joint stiffness and pain, and cosmetic appearance. The presence of complications apart from recurrent HV was also an indicator (including cock-up deformity, transfer metatarsalgia, hallux varus, nonunion). Only one study provided patient level data about reasons for revision. Out of the 301 feet, we could confirm that 262 (87%) had recurrent HV leading to revision surgery. The remaining 39 patients had other reasons for revision surgery such as hallux varus or transfer metatarsalgia.

### Outcomes

Various methods were used in assessment of outcomes in all 10 included studies, the most common of these are summarized in Table 3 . The American Orthopaedic Foot and Ankle Society (AOFAS) score was a feature in every outcome assessment. Only 2 studies did not have preoperative scores for comparison postop. Improvement in AOFAS score was an average of 38.3 for those undergoing MIS revisions, compared to 26.8 in those using traditional open techniques. Other measures used by the studies included pain scoring and patient satisfaction. HVA and/or IMA were used in measurements of pre - and postop radiographs in all 10 studies, respectively. Revision approaches using MIS techniques showed median postoperative reduction in IMA and HVA of 5.6 and 18.4 degrees, respectively, compared to a median of 4.4 and 15.5 degrees, respectively, for open revision techniques. Overall, metrics varied widely between studies including other less specific measures such as pain scores and satisfaction rates, hence preventing pooling of outcomes.

**Table 3:** Outcomes measured in patients for included studies

Article (year)	Revision approach	No. of patients (feet)	AOFAS score (pre- to postop)	Pain score (pre- to postop)	IMA ° (pre- to postop)	HVA ° (pre- to postop)	DMAA ° (pre- to postop)	Weight-bearing status
Magnan et al (2019)	MIS – distal first metatarsal osteotomy	26 (32)	46.9 to 85.2	NA	11.4 to 6.6	26.1 to 10.5	15.2 to 7.3	Immediate full weight bearing
Scala et al (2020)	MIS – MSMO	52 (54)	48 to 86	NA	14 to 8	30 to 10	18 to 12	Weight bearing as tolerated for 2 weeks with RICE
Ellington et al (2011)	Open – Lapidus procedure	23 (25)	82.8 postop (preop not done)	2.4 postop (preop not recorded) (VAS scoring)	13.6 to 7.5	36.2 to 15.2	18.6 to 11.7	Immediate full weight bearing
Bock et al (2010)	Open – Scarf osteotomy	35 (39)	56.2 to 89.9	5.9 to 0.4 (VAS scoring)	13.1 to 4.3	30 to 7.7	15 to 8	Immediate full weight bearing
Grimes and Coughlin (2006)	Open – First MTP joint arthrodesis	29 (33)	73 postop (preop not done)	NA	9 to 8	23 to 16	NA	Not specified
Kannegieter and Kilmartin (2011)	Open – Combined reverse scarf and opening wedge osteotomy	5 (NA)	89 to 74	NA	5 to 8.9	NA	NA	Not specified
Rose et al (2014)	Open – Scarf osteotomy	31 (36)	49.6 to 83.4	NA	12.9 to 8.9	27.7 to 13.6	NA	Not specified
Vienne et al (2006)	Open – First MTP joint arthrodesis	20 (22)	44 to 85	18 to 37 (score system not specified)	NA	24 to 16	NA	Immediate full weight bearing
Coetzee et al (2003)	Open – Lapidus procedure	24 (26)	47.6 to 76.7	6.2 to 3 (VAS scoring)	18 to 7.6	37.1 to 14.8	NA	Nonweight bearing for 6 weeks
Machacek et al (2004)	Open – First MTP joint arthrodesis	28 (29)	42 to 80	NA	9.9 to 9.8	27.3 to 14.5	NA	Weight bearing as tolerated 4–6 weeks

Abbreviations: AOFAS, American Orthopaedic Foot and Ankle Society; DMAA, distal metatarsal articular angle; HVA, hallux valgus angle; IMA, intermetatarsal angle; MIS, minimally invasive surgical; MSMO, modified subcapital metatarsal osteotomy; MTP, metatarsophalangeal; RICE, rest, ice, compression, and elevation; VAS, Visual Analog Scale.

In the included studies, postoperative protocols (including weight-bearing status and use of walking aids) largely varied. Of the studies including this data, 7 studies included information about weight bearing—57.1% (4) recommended immediate full weight-bearing postop, 28.6% (2) weight bearing as tolerated for 6 weeks, and 14.3% (1) nonweight bearing for up to 6 weeks. Of note, both MIS revisions papers recommended early weight-bearing status which might in part account for some of the improved outcomes. 25 Follow-up period following revision surgery also differed greatly, which in turn would have affected the time at which postoperative outcome scores were measured. Follow-up of patients in the MIS revision papers ranged between 1 and 15.2 months. In the papers featuring traditional techniques, it ranged between 1 and 89 months.

### Complications

Overall complication rates following revision HV surgery for patients are summarized in Table 4 . Nine of the 10 included studies highlighted complications in patients following revision surgery. Of the 301 feet, 74 complications were reported. Complications reported included nonunion (13–4.3%), painful or broken metalwork requiring correction/removal (13–4.3%), infection requiring antibiotics (7–2.3%), and transfer metatarsalgia (11–3.7%). Others included recurrence HV following revision (3 [1%]), delayed union (3 [1%]), malunion (3 [1%]), stiffness (3 [1%]), and hallux varus (3 [1%]). No cases of avascular necrosis or further stress fractures were noted as complications in the included studies.

**Table 4:** Overall complication rates following revision hallux valgus surgery for patients/feet in included studies

Complications	Number of cases (percentage of total feet in included studies)
Recurrent hallux valgus	3 (1)
Infection	7 (2.3)
Delayed union	3 (1)
Non union	13 (4.3)
Malunion	3 (1)
Stiffness	3 (1)
Reduction in range of motion (ROM)	2 (0.7)
Transfer metatarsalgia	11 (3.7)
Painful/broken metalwork	13 (4.3)
Miscellaneous (including loosening of implant, generalized discomfort, paresthesia)	12 (4)

## Discussion

The results show promise over a range of techniques including both open and MIS techniques when applied in revision surgery. Most studies demonstrated improvements in both quantitative as well as qualitative outcomes. However, any firm recommendations for MIS over open techniques are limited by the inability to pool outcomes and directly compare studies. This was also partly due to the evidence levels of studies as well as heterogeneity in demographics, indications for surgery, and outcome measures. The evidence for MIS techniques in the revision setting is sparse compared with techniques described for primary correction of HV. 6 In time, it is anticipated that newer MIS techniques, such as third-generation minimally invasive Chevron–Akin osteotomy may be described in the revision setting as surgeons become more comfortable with the procedure. The current literature base also lacks consistent information on patient comorbidities which would have been contributive factors in determining which patients were more suitable for MIS versus open techniques.

In addition to the limited evidence levels, the main limitation preventing firm recommendations of MIS over open is the widely varying operative techniques used. Previous studies have described percutaneous minimally invasive correction of HV using a burr. 8 Magnan et al appears to use a similar percutaneous technique, while Scala et al uses a mini open technique (the MSMO osteotomy) applying a microsagittal saw, thereby limiting our ability to compare related outcomes.

The AOFAS score was featured in each study allowing limited comparison without accounting for the heterogeneity in study participants and protocols. AOFAS is the most commonly used PROM in foot and ankle surgery, 26 but its validity has been questioned. 27 28 Currently, this is the best comparator for the included studies, but it is hoped that future research might also include other metrics which have shown greater validity, such as the Manchester–Oxford Foot Questionnaire. 29

In view of these limitations and variation depending on the index procedure performed, literature comparing across different techniques used in primary HV surgery 30 has found a mean improvement of AOFAS score by 32.9 postoperatively. This was more similar to the scores achieved using MIS compared to open techniques in revision HV surgery highlighted earlier. For quantitative outcomes using radiological measurements, a third of our included studies also included measurement of DMAA—while featured in our results, this was not used as a basis for comparison due to poor intra- and interobserver reliability noted in previous studies. 31 Use of IMA and HVA have been confirmed previously as criterion standard in outcome measurement in HV surgery, 32 33 with newer studies even confirming accuracy in use beyond radiographic imaging through digital and mobile imaging. 34 35 In primary HV correction, a preoperative HVA of 40 degrees and above as well as an immediate postoperative HVA of 8 degrees and above have been significantly associated with HV recurrence. 11 Extrapolating this with outcomes above, the latter was only achieved in less than 19 (6.3%) of total feet corrected (using open techniques), thereby highlighting scope for improvement in both MIS and open techniques mentioned for revision HV in reducing re-recurrence. Length of follow-up and in turn time taken for the postoperative outcome scores to be measured also varied widely between the studies—evidence has shown that this can have a statistically significant impact, with worsening of scores over time. 36

Another limitation of this study was the heterogeneity of the current literature base. Some paper listed subjective exclusion criteria including severe degenerative changes, significant stiffness, and instability, whereas others used subjective measurements of radiographic parameters. Time between the primary and revision surgery was also not specified, limiting our ability to draw comparisons or possible correlations between this factor and complication rates, or if there had been adequate trial of conservative management options beforehand. Regarding the revision rates, none of the studies had in fact made reference to the total number of feet/patients who had primary HV procedures from which they then included in their studies after follow-up upon consideration for revision HV surgery, hence it was impossible to extrapolate this information.

This study aimed to identify patients who had revision surgery for recurrent HV alone, rather than for other complications following primary HV correction. Based on the data available it was not possible to differentiate between the patient groups, with all results pooled together for analysis of outcomes. When screening the references of papers in the literature search, four additional older articles were found which described the open treatment of recurrent HV. They were published before the time period specified in the search strategy. Given the small patient numbers featured (between 9 and 16 patients) and limited access, it was felt that the data derived from them would not have significantly affected our findings.

## Conclusion

This article has demonstrated current outcomes related to HV revision surgery. MIS techniques grouped did not show worse outcomes or safety concerns compared to open techniques. There is a paucity in the literature of modern MIS techniques for treatment of recurrent HV. Exploring and developing the percutaneous methods in MIS techniques for revision HV surgery provides an exciting possibility for future work moving forward.
